# Thinking About Reasons for One’s Choices Increases Sensitivity to Moral Norms in Moral-Dilemma Judgments

**DOI:** 10.1177/01461672231180760

**Published:** 2023-06-29

**Authors:** Nyx L. Ng, Dillon M. Luke, Bertram Gawronski

**Affiliations:** 1 The University of Texas at Austin, USA

**Keywords:** CNI model, dual-process model, moral-dilemma judgment, reasons, reflection

## Abstract

Whereas norm-conforming (deontological) judgments have been claimed to be rooted in automatic emotional responses, outcome-maximizing (utilitarian) judgments are assumed to require reflective reasoning. Using the CNI model to disentangle factors underlying moral-dilemma judgments, the current research investigated effects of thinking about reasons on sensitivity to consequences, sensitivity to moral norms, and general action preferences. Three experiments (two preregistered) found that thinking about reasons (vs. responding intuitively or thinking about intuitions) reliably increased sensitivity to moral norms independent of processing time. Thinking about reasons had no reproducible effects on sensitivity to consequences and general action preferences. The results suggest that norm-conforming responses in moral dilemmas can arise from reflective thoughts about reasons, challenging the modal view on the role of cognitive reflection in moral-dilemma judgment. The findings highlight the importance of distinguishing between degree (high vs. low elaboration) and content (intuitions vs. reasons) as distinct aspects of cognitive reflection.

A major debate in moral psychology concerns the role of cognitive reflection in moral judgment. Whereas early rationalist theories regarded moral judgments as the product of reflective reasoning processes (e.g., Kohlberg, 1976), recent intuitionist approaches emphasize the role of automatic emotional processes in moral judgments (Greene & Haidt, 2002). Integrating both ideas, the dual-process model (DPM) of moral-dilemma judgment holds that characteristically utilitarian judgments (i.e., judgments maximizing the greater good) arise from reflective reasoning about costs and benefits, whereas characteristically deontological judgments (i.e., judgments conforming to moral norms) are driven by automatic emotional reactions to the idea of causing harm (Greene, 2007).

In the current work, we investigated the effects of a particular type of reflection process on moral-dilemma judgments: thinking about reasons for one’s choices. In line with the definition provided by Merriam-Webster’s Dictionary, we conceptualize *reasons* as statements that justify and explain a belief or action. Although the reasons people use to justify their choices can come in myriad forms, people are especially likely to think about reasons when they anticipate having to explain their choices to others (Lerner & Tetlock, 1999). Prior research outside of the moral domain suggests that introspecting on reasons for one’s preferences can shape judgments and decisions in fundamental ways (Wilson et al., 1989). Using a formal modeling approach to disentangle different factors underlying moral-dilemma judgments (Gawronski et al., 2017), the current studies expand on these findings by investigating how thinking about reasons for one’s choices influences sensitivity to consequences, sensitivity to moral norms, and general preference for inaction versus action in moral-dilemma judgments.

## Cognitive Reflection and Moral-Dilemma Judgment

The DPM posits that situations wherein maximizing the greater good conflicts with adherence to moral norms (e.g., a situation where killing one would save the lives of multiple others) automatically elicit deontological intuitions, which may be overridden in favor of utilitarian judgments via reflective reasoning (Greene, 2007). Research supportive of this idea has demonstrated that situational disruption of reflective reasoning interferes with utilitarian judgments (e.g., Greene et al., 2008; Suter & Hertwig, 2011) and that individuals with a stronger propensity to engage in reflective reasoning are more likely to make utilitarian judgments (e.g., Patil et al., 2021).

However, a closer inspection of relevant findings reveals that the available evidence regarding the proposed link between cognitive reflection and moral judgments is rather mixed. Among studies that experimentally manipulated cognitive reflection through time pressure, some have found that time pressure (a) attenuated utilitarian judgments (Körner & Volk, 2014; Suter & Hertwig, 2011), (b) increased utilitarian judgments (Hashimoto et al., 2022), or (c) had no effect on moral-dilemma judgments (Gürçay & Baron, 2017; Tinghög et al., 2016). Yet, other studies found that effects of time pressure depend on contextual factors, such as whether cost–benefit ratios were “efficient” (kill one to save 500) versus “inefficient” (kill one to save five; Trémolière & Bonnefon, 2014) or whether participants thought about moral dilemmas in concrete versus abstract terms (Körner & Volk, 2014). Using a process dissociation approach to disentangle utilitarian and deontological tendencies (see Conway & Gawronski, 2013), McPhetres et al. (2018) found that time pressure reduced deontological tendencies among religious individuals and that time pressure had no effect on utilitarian tendencies. Research that experimentally manipulated cognitive reflection through cognitive-load tasks have produced similarly mixed findings. Whereas Körner and Volk (2014) found that participants who thought about moral dilemmas in concrete rather than abstract terms made more deontological judgments when they were under cognitive load, other studies revealed no effects of cognitive load on moral judgments (Tinghög et al., 2016). On an individual-difference level, some studies suggest that tendencies to engage in reflective reasoning are positively associated with utilitarian judgments (Baron et al., 2015; Patil et al., 2021; Paxton et al., 2012). However, in studies that teased apart utilitarian and deontological tendencies via process dissociation (see Conway & Gawronski, 2013), cognitive reflection has been found to be positively associated with both utilitarian and deontological tendencies (Byrd & Conway, 2019). Together, these results suggest that the relation between cognitive reflection and moral-dilemma judgments is more complex than suggested by the DPM.

## Thinking About Reasons

A central characteristic of experimental studies examining the role of cognitive reflection in moral-dilemma judgments is that they focused predominantly on effects of processing resources (e.g., time pressure, cognitive load). Although this approach is helpful for understanding the amount of resources required by the process underlying a particular type of judgment, it remains silent about the specific contents of that process. Shifting the focus from processing resources to mental contents, the current work investigated how thinking about reasons for one’s choices influences moral-dilemma judgments. Research suggests that introspecting on reasons for one’s preferences influences product choices (Wilson et al., 1993), post-choice satisfaction (Wilson et al., 1993), predictions about how one would act in a situation (Wilson & LaFleur, 1995), and attitude-behavior consistency (Wilson et al., 1984). These findings raise interesting questions about how thinking about reasons might influence moral judgments.

What role might reasons play in moral judgments? Some theories suggest that the reasons people generate for their moral judgments are mere post hoc rationalizations of moral intuitions whose origins are outside of awareness (Haidt, 2001). According to this view, thinking about reasons should have little impact on moral judgments, because thoughts about reasons are not causally involved in producing moral judgments; they are mere afterthoughts to moral intuitions arising from unconscious processes. Yet, different from these assumptions, the DPM implies the possibility that, although the reasons generated for deontological judgments might be mere post hoc rationalizations of unconsciously generated moral intuitions, thinking about reasons may enhance utilitarian judgments by promoting cost–benefits analyses. According to this view, thinking about reasons should influence moral-dilemma judgments in a manner similar to the presumed effect of processing resources. However, as noted above, the available evidence regarding the latter idea is rather mixed. Moreover, effects of thinking about reasons on moral-dilemma judgments have, to date, only been studied in a post-decision context. In a series of studies, Stanley et al. (2018) instructed participants to respond to a moral dilemma and then evaluate reasons supporting either their chosen option, rejected option, or both. The studies revealed that evaluating reasons after a decision had already been made rarely induced changes in participants’ decisions, regardless of whether the reasons affirmed or opposed their decisions. While Stanley et al.’s findings are informative about the ineffectiveness of thinking about reasons in changing moral decisions that have already been made (akin to the notion of post hoc rationalization; see Haidt, 2001), it remains unclear whether and how thinking about reasons before one decides may influence moral judgments.

## The Current Research

The main goal of the current research was to address the questions of whether and how thinking about reasons before making a decision influences moral-dilemma judgments. To gain more nuanced insights, we utilized the CNI model (Gawronski et al., 2017) to resolve two confounds in the traditional dilemma paradigm. The two confounds can be illustrated with the classic trolley problem, in which a runaway trolley is hurtling toward five people who would be killed unless the trolley is redirected to another track where it would kill only person (i.e., switch dilemma; see Foot, 1967) or a large man is pushed from a bridge in front of the trolley to stop it (i.e., footbridge dilemma; see Thomson, 1985). Judgments supporting these actions have been interpreted as characteristically utilitarian in the sense that they maximize the greater good (i.e., killing one saves the lives of five; see Conway et al., 2018). Judgments opposing the described actions have been interpreted as characteristically deontological in the sense that they conform to a relevant moral norm (i.e., the moral norm that prohibits the killing of other people; see Conway et al., 2018).

A major problem with the trolley dilemma and structurally similar scenarios is that they include two confounds that create ambiguities in the interpretation of empirical results. First, by pitting utilitarian against deontological judgments, endorsement of the utilitarian option involves rejection of the deontological option and vice versa, which leads to ambiguities about a whether a given finding is driven by the process underlying utilitarian judgments, the process underlying deontological judgments, or both (Conway & Gawronski, 2013). Second, by conflating utilitarian judgments with action and deontological judgments with inaction, the traditional dilemma paradigm conflates adherence to moral doctrines with general action tendencies (Crone & Laham, 2017).

**Figure 1.**
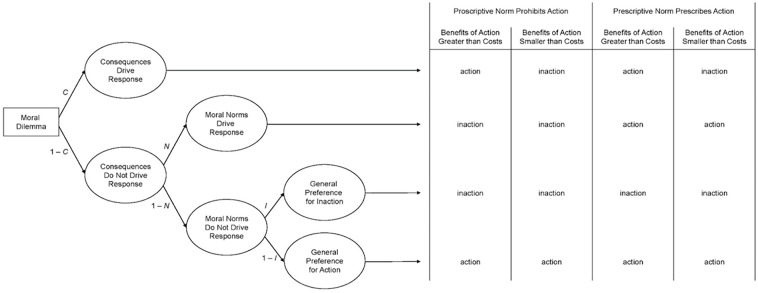
CNI Model of Moral Decision-Making Predicting Action versus Inaction Responses in Moral Dilemmas With Proscriptive and Prescriptive Norms, and Consequences Wherein the Benefits of Action Are Either Greater or Smaller Than the Costs of Action. *Source.* Reproduced from Gawronski et al. (2017). Reprinted with permission from the American Psychological Association.

The two confounds can be resolved with the CNI model of moral judgment and decision-making, a multinomial model that separately quantifies sensitivity to consequences (*C*), sensitivity to moral norms (*N*), and general preference for inaction versus action (*I*) in responses to moral dilemmas (Gawronski et al., 2017). The three model parameters are estimated using responses to four types of moral dilemmas that differ in terms of cost–benefit ratios (i.e., benefits associated with action are either greater or smaller than costs) and type of focal norm (i.e., proscriptive or prescriptive). The CNI model’s *C* parameter captures the extent to which participants’ responses to moral dilemmas are sensitive to consequences such that they (a) support action when the benefits associated with action outweigh their costs and (b) support inaction when the costs outweigh the benefits (first row in Figure 1). The *C* parameter reflects the difference between these two specific cases. Although the *C* parameter could be argued to reflect the general norm *always maximize the benefits* (Hennig & Hütter, 2020), the response pattern captured by the *C* parameter is distinct from the one captured by the CNI model’s *N* parameter on sensitivity to moral norms, which reflects the extent to which participants (a) support action when the action is prescribed by a prescriptive norm and (b) support inaction when the action is prohibited by a proscriptive norm (second row in Figure 1). Instead of presuming that the response pattern captured by the *N* parameter is driven by conscious thoughts about specific moral norms, the *N* parameter simply captures the difference in responses between cases wherein the action either causes or prevents proximal harm. Finally, the CNI model’s *I* parameter captures the extent to which participants’ responses reflect a general preference for inaction versus action such that participants support inaction (vs. action) regardless of cost–benefit ratios and types of moral norm (third and fourth rows in Figure 1). Although the response pattern captured by the *I* parameter may be argued to reflect the general norm *first, do no harm* (Baron & Goodwin, 2020), this response pattern is again distinct from the one captured by the *N* parameter. Whereas the *N* parameter captures discrepancies between moral dilemmas wherein the focal action either causes or prevents proximal harm, the *I* parameter captures general preferences for inaction (vs. action) regardless of whether the focal action causes or prevents proximal harm and regardless of cost–benefit ratios. Adherence to the general norm *first, do no harm* would be reflected in a general preference for inaction regardless of the moral-dilemma variant. The CNI model disentangles sensitivity to consequences, sensitivity to moral norms, and general preference for inaction versus action in responses to moral dilemmas by quantifying these three distinct response patterns.

Research by Luke and Gawronski (2021) found that the *C* and *N* parameters had high reliability in terms of their internal consistency (Cronbach’s αs > .69) and test–retest correlations (*r*s > .80), whereas reliability estimates obtained for the *I* parameter were lower for both internal consistency (Cronbach’s αs between .37 and .53) and test–retest stability (*r* = .41). Like research using the traditional trolley paradigm, research utilizing the CNI model has generated mixed results on the effects of processing resources on moral-dilemma judgments. On one hand, two studies by Gawronski et al. (2017) found that cognitive load induced through a cognitively taxing memorization task (versus an easier memorization task) increased general preference for inaction without affecting sensitivity to consequences and sensitivity to moral norms. On the other hand, Kroneisen and Steghaus (2021) found that time pressure reduced sensitivity to consequences without affecting general preference for inaction and sensitivity to moral norms. In a follow-up study, however, Kroneisen and Steghausen (2021) failed to obtain a significant effect of deliberation instructions on sensitivity to consequences and instead found a significant positive association between individual differences in reaction times and sensitivity to moral norms. Together, these results echo concerns that effects of processing resources on moral-dilemma judgments are mixed and inconclusive.

Shifting the focus from processing resources to mental contents, the current research investigated how thinking about reasons for one’s choices influences moral-dilemma judgments. Toward this end, participants in the current studies were asked to respond to a battery of moral dilemmas developed for research using the CNI model (Körner et al., 2020). In the first two experiments, half of the participants were instructed to think about reasons for their choices before making a judgment (*think-about-reasons condition*). The remaining half was asked to rely on their spontaneous “gut” reactions when making a judgment (*rely-on*-*intuitions condition*). In the third experiment, participants were instructed to either rely on their spontaneous “gut” reactions when responding to the moral dilemmas (*rely-on*-*intuitions condition*), to take a moment to think about their intuitions before responding to the moral dilemmas (*think-about-intuitions condition*), or to take a moment to think about reasons that would justify their responses before responding to the moral dilemmas (*think-about*-*reasons condition*). Responses in all three studies were analyzed using the CNI model to disentangle effects on sensitivity to consequences, sensitivity to moral norms, and general preference for inaction versus action. For each experiment, we report how we determined our sample size, all data exclusions, all manipulations, and all measures. All data, analysis codes, and materials are available at https://osf.io/cyvb7/.

## Experiment 1

Experiment 1 was conducted as an exploratory study to investigate whether thinking about reasons influences sensitivity to consequences, sensitivity to moral norms, and general preference for inaction versus action in moral-dilemma judgments.

### Method

#### Participants

Participants were recruited via Amazon’s Mechanical Turk (MTurk). Participation eligibility criteria were as follows: (a) located in the United States, (b) at least 18 years old, (c) approved for at least one previous assignment, (d) minimum approval rate of 95% on past assignments, and (e) no prior participation in studies from the authors’ lab involving the same moral-dilemma battery. We aimed to recruit 200 participants (100 per condition), which provides a statistical power of 80% in detecting a small-to-medium sized difference of *d* = 0.40 between two independent means, α= (two-tailed). Participants were compensated $4.00.

Of the 217 MTurk workers who started the study, 16 did not complete it, 25 failed a first attention check, and another 11 failed a second attention check. Data from these participants were excluded from the analyses, resulting in a final sample of 165 participants (*n*_rely-on-intuitions_ = 83; *n*_think-about-reasons_ = 82). Participant demographics were as follows: 43.0% female, 57.0% male; *M*_age_ = 33.1 years; 80.6% White, 9.7%, Black/African American, 8.5% Asian, 4.8% other races; 13.3% Spanish/Hispanic/Latino; 89.1% had at least attended some college; 46.7% identified as being politically liberal, 23.6% as neither liberal nor conservative, and 29.7% as conservative.

#### Procedure

After providing informed consent, participants were randomly assigned to either the think-about-reasons condition or rely-on-intuitions condition. Participants in both conditions were told that they will be presented with a series of short stories and that they will be asked to make a judgment about whether they find the action described acceptable. Participants in the think-about-reasons condition received the following instructions:

*For each story, we ask that you think very carefully about the options when making your decision. As you read through the stories and make your judgments, you should think about reasons that justify your responses. At the end of the study, you will be asked to justify some of your decisions and explain your reasoning.*

Participants in the rely-on-intuitions condition were given the following instructions in addition to the basic instructions for the moral-judgment task:

*For each story, we ask that you go with your first intuitive reaction when making your decision. As you read through the stories and make your judgments, you should trust your intuitions and use your spontaneous “gut” reactions. You do not have to justify your decisions or explain your reasoning.*

Next, all participants completed a battery of 48 moral dilemmas adapted from Körner et al. (2020), following which only those in the think-about-reasons condition were prompted to justify their responses on five randomly selected dilemmas. The 48 moral dilemmas comprised 12 basic scenarios in four variants, all of which were based on real-world events. Each dilemma was accompanied by a binary *yes*/*no* question asking participants to indicate whether the described action was acceptable. At the end of the study, participants completed a set of demographic questions and two attention checks. Demographic questions answered comprised gender, age, ethnicity, race, education, and political ideology. The first attention check was a reading-intensive item (Oppenheimer et al., 2009) that instructed participants to not select any options but instead skip ahead to the next screen. Participants who selected any of the response options were thus considered to have failed the attention check. The second attention check comprised three items, with the first requiring participants to type out in capital letters the sentence *Dan went to the store to buy fruit;* the second asking participants to answer the question *Where did Dan likely go?* with *grocery store*, *clothing store*, and *furniture store* as response options; and the third asking participants to answer the question *What did Dan buy?* with *a type of food*, *a pair of shoes*, and *a piece of furniture* as response options. Participants who did not accurately respond to the three items were considered to have failed the second attention check.

#### Data Analysis

The CNI model was fitted to the data to estimate separate *C*, *N*, and *I* parameters for each condition. Modeling analyses were conducted with the freeware multiTree (Moshagen, 2010) using the template files for CNI model analyses provided by Gawronski et al. (2017) at https://osf.io/m82k7/. Following Gawronski et al. (2017), we used a fixed estimation algorithm with random start values, two replications, and a maximum of 90,000 iterations. Likewise, following Gawronski et al. (2017), Cohen’s *d* effect sizes of between-group differences were calculated with Lipsey and Wilson’s (2001) online companion to meta-analyses at https://www.campbellcollaboration.org/escalc/html/EffectSizeCalculator-SMD8.php using means, standard errors, and sample sizes.

### Results

#### Traditional Dilemma Analysis

To permit a comparison of our findings to research using the traditional dilemma paradigm, we explored whether thinking about reasons influences responses to dilemmas wherein the focal action is prohibited by a proscriptive norm and action leads to better outcomes for the greater good (equivalent to the structure of the trolley problem). Toward this end, we compared the mean proportions of responses endorsing action (*yes*) versus inaction (*no*) on dilemmas of this type (i.e., traditional score) across the two conditions. Higher scores on the traditional score can be interpreted as reflecting a greater relative preference for utilitarian over deontological judgments (see Conway et al., 2018). Thinking about reasons did not have a significant effect on relative preference for utilitarian over deontological judgments, *t*(163) = 0.47, *p* = .643, *d* = 0.073.

#### CNI Model Analysis

The CNI model fit the data well, *G*^2^(2) = 2.17, *p* = .337, *w* = 0.017. Means and 95% confidence intervals of the estimated parameter scores are presented in Table 1. The *N* parameter significantly differed across conditions, Δ ∆*G*^2^(1) = 11.66, *p* < .001, *d* = 0.535, indicating that participants in the think-about-reasons condition showed a significantly stronger sensitivity to moral norms than those in the rely-on-intuitions condition. The *I* parameter was marginally different across conditions, Δ ∆*G*^2^(1) = 2.94, *p* = .086, *d* = 0.269, indicating that participants in the think-about-reasons condition tended to show a greater general preference for action compared with participants in the rely-on-intuitions condition. The *C* parameter did not significantly differ across conditions, Δ ∆*G*^2^(1) = 0.53, *p* = .465, *d* = 0.114.

**Table 1:** Means and 95% Confidence Intervals of Moral Dilemma Action (vs. Inaction) Indices, and CNI Model Parameters, Experiment 1.

	Full sample (*N =* 165)				Reduced sample (*N =* 154)			
	Rely on intuitions (*n* = 83)		Think about reasons (*n* = 82)		Rely on intuitions (*n* = 80)		Think about reasons (*n* = 74)	
	*M*	95% CI	*M*	95% CI	*M*	95% CI	*M*	95% CI
Moral-dilemma indices								
Proscriptive norm prohibits action								
Benefits of action > costs	5.27	[4.71, 5.82]	5.06	[4.39, 5.73]	5.18	[4.61, 5.74]	4.97	[4.26, 5.68]
Benefits of action < costs	3.11	[2.49, 3.73]	2.77	[2.05, 3.49]	3.03	[2.39, 3.66]	2.69	[1.94, 3.44]
Prescriptive norm prescribes action								
Benefits of action > costs	9.39	[9.00, 9.77]	9.82	[9.36, 10.27]	9.45	[9.06, 9.84]	9.80	[9.30, 10.30]
Benefits of action < costs	6.72	[6.19, 7.25]	7.63	[7.07, 8.20]	6.80	[6.26, 7.34]	7.57	[6.96, 8.17]
CNI model parameters								
*C* parameter	0.20	[0.17, 0.23]	0.19	[0.16, 0.21]	0.20	[0.17, 0.23]	0.19	[0.16, 0.22]
*N* parameter	0.40	[0.37, 0.44]	0.49	[0.46, 0.53]	0.42	[0.38, 0.46]	0.50	[0.46, 0.54]
*I* parameter	0.48	[0.45, 0.51]	0.44	[0.40, 0.47]	0.48	[0.45, 0.51]	0.45	[0.41, 0.48]

*Note.* Full sample refers to the data before exclusion of response-time outliers; reduced sample refers to the data after exclusion of response-time outliers. Moral-dilemma indices scores can range from 0 to 12. CNI model parameter scores can range from 0 to 1. CI = confidence interval.

#### Response-Time Analysis

To explore whether the obtained effects on moral judgments are related to differences in the resources participants devoted to thinking about their responses, we created an index of response time by calculating the total time participants spent on the moral dilemmas. Response times were marginally longer in the think-about-reasons condition compared with the rely-on-intuitions condition (*M*_think-about-reasons_ = 1546.02; *M*_rely-on-intuitions_ = 1235.60), *t*(126.64) = −1.97, *p* = .051, *d* = 0.301. To rule out undue effects of response-time outliers, we computed the median absolute deviation (*MAD* = 700.87) for total response time and applied a moderately conservative criterion in which cases are identified as outliers if they lie beyond 2.5 absolute deviations from the median (Leys et al., 2013). Using this procedure, 11 cases were flagged as potential outliers (*n*_rely-on-intuitions_ = 3; *n*_think-about-reasons_ = 8). After excluding these outliers, response times did not significantly differ across conditions (*M*_think-about-reasons_ = 1227.22; *M*_rely-on-intuitions_ = 1163.35), *t*(152) = −0.61, *p* = .545, *d* = 0.098. The CNI model still fit the data well with the reduced sample, *G*^2^(2) = 1.99, *p* = .370, *w* = 0.016. Moreover, consistent with the results obtained with the full sample, participants in the think-about-reasons condition showed a significantly greater sensitivity to moral norms than participants in the rely-on-intuitions condition, Δ ∆*G*^2^(1) = 8.44, *p* = .004, *d* = 0.472, but there were no significant differences in the *C* parameter, Δ ∆*G*^2^(1) = 0.35, *p* = .557, *d* = 0.095, and *I* parameter, Δ ∆*G*^2^(1) = 1.37, *p* = .241, *d* = 0.191.

### Discussion

Although the traditional dilemma analysis revealed no significant effect of thinking about reasons, the results of CNI model analyses suggest that thinking about reasons increases sensitivity to moral norms without affecting sensitivity to consequences and general action tendencies. These findings conflict with the ideas that (a) the reasons generated for norm-conforming judgments are mere post hoc rationalizations of unconsciously generated moral intuitions and (b) thinking about reasons enhances effects of costs and benefits for the greater good. If the reasons generated for norm-conforming judgments are mere post hoc rationalizations of unconsciously generated moral intuitions, moral-dilemma judgments should not differ depending on whether participants are asked to think about reasons or rely on their intuitions. Moreover, if thinking about reasons enhances effects of costs and benefits for the greater good, thinking about reasons should increase sensitivity to consequences without affecting sensitivity to moral norms.

Another interesting finding is that thinking about reasons influenced moral judgments independent of the overall time participants spent thinking about their responses. Although participants in the think-about-reasons condition tended to spend more time on the moral-judgment task compared with participants in the rely-on-intuitions condition, this difference disappeared when response-time outliers were removed from the analyses. Yet, removing response-time outliers did not qualify the obtained effect on moral judgments, in that thinking about reasons continued to increase sensitivity to moral norms without affecting sensitivity to consequences. Together, these results suggest that the obtained differences in moral judgments were driven by the contents of participants’ thought processes (i.e., reasons vs. intuitions) instead of the amount of invested resources.

## Experiment 2

Experiment 2 sought to replicate the findings of Experiment 1 in a preregistered lab study with the same materials. The study was formally preregistered at https://osf.io/7sh3w/.

### Method

#### Participants

We preregistered to recruit 250 participants for Experiment 2. The sample size decision was based on a power analysis using G*Power 3.1 (Faul et al., 2007), aiming for at least 80% power in detecting a between-group difference of *d* = 0.36 with an alpha level of .05 (two-tailed).^1^ Participants were recruited from a pool of undergraduate students enrolled in an introductory psychology course. Due to an administrative error by the research assistants who ran the study, 257 participants completed the study. To be consistent with our preregistered stopping rule, those who started the study after the first 250 participants had been approved for credit were excluded from analyses. Following our preregistered exclusion criteria, data from one participant who did not complete all moral dilemmas were excluded from analyses. The final sample size for the second study was thus *N* = 249 (*n*_rely-on-intuitions_ = 128; *n*_think-about-reasons_ = 121).^2^ Participant demographics are as follows: 67.9% female, 31.7% male, 0.4% others; *M*_age_ = 19.3 years; 59.0% White, 6.4% Black/African American, 32.1% Asian, 7.6% other races; 26.5% Spanish/Hispanic/Latino; 100% of them were high-school graduates given that they were recruited from an undergraduate participant pool; 61.0% of them identified as being politically liberal, 19.7% as neither liberal nor conservative, and 19.3% as conservative.

#### Procedure and Measures

Experiment 2’s procedure was identical to that of Experiment 1 with three exceptions. First, instead of completing the study online on Qualtrics, participants reported to a psychological laboratory, were seated in individual testing rooms, and completed the assessment on MediaLab. Second, because the study was run in-person in the authors’ lab, attention checks were not included in the second study. Third, participants received research credit for an introductory psychology course instead of monetary compensation.

### Results

#### Exploratory Traditional Dilemma Analysis

Consistent with Experiment 1, relative preference for utilitarian over deontological judgments did not significantly differ across experimental conditions, *t*(247) = 0.26, *p* = .797, *d* = 0.033.

#### Preregistered CNI Model Analysis

The CNI model fit the data well, *G*^2^(2) = .056, *p* = .972, *w* = 0.002. Means and 95% confidence intervals of the estimated parameter scores are presented in Table 2. Consistent with the results of Experiment 1, preregistered confirmatory analyses revealed that participants in the think-about-reasons condition showed a stronger sensitivity to moral norms than those in the rely-on-intuitions condition, Δ ∆*G*^2^(1) = 8.05, *p* = .005, *d* = 0.362. Inconsistent with the null effect on the *C* parameter in Experiment 1, participants in the think-about-reasons condition showed a significantly stronger sensitivity to consequences than their counterparts in the rely-on-intuitions condition, Δ ∆*G*^2^(1) = 4.10, *p* = .043 *d* = 0.258. A significant difference was also found for the *I* parameter, Δ ∆*G*^2^(1) = 4.41, *p* = .036, *d* = 0.269. However, the direction of the obtained effect was opposite to the marginal effect in Experiment 1, in that participants in the think-about-reasons condition showed a greater preference for inaction than those in the rely-on-intuitions condition.^3^

**Table 2.** Means and 95% Confidence Intervals of Moral Dilemma Action (vs. Inaction) Indices, and CNI Model Parameters, Experiment 2.

	Full sample (*N =* 249)				Reduced sample (*N =* 243)			
	Rely on intuitions (*n* = 128)		Think about reasons (*n* = 121)		Rely on intuitions (*n* = 126)		Think about reasons (*n* = 117)	
	*M*	95% CI	*M*	95% CI	*M*	95% CI	*M*	95% CI
Moral-dilemma indices								
Proscriptive norm prohibits action								
Benefits of action > costs	5.24	[4.78, 5.70]	5.16	[4.69, 5.62]	5.23	[4.77, 5.69]	5.22	[4.75, 5.69]
Benefits of action < costs	1.76	[1.48, 2.03]	1.26	[1.01, 1.51]	1.78	[1.50, 2.06]	1.30	[1.04, 1.56]
Prescriptive norm prescribes action								
Benefits of action > costs	10.01	[9.72, 10.30]	10.19	[9.96, 10.42]	10.00	[9.72, 10.30]	10.15	[9.91, 10.38]
Benefits of action < costs	6.46	[6.04, 6.88]	6.29	[5.86, 6.71]	6.44	[6.01, 6.86]	6.27	[5.84, 6.70]
CNI model parameters								
*C* parameter	0.29	[0.27, 0.31]	0.32	[0.30, 0.35]	0.29	[0.27, 0.31]	0.32	[0.30, 0.35]
*N* parameter	0.56	[0.53, 0.59]	0.62	[0.59, 0.65]	0.56	[0.53, 0.59]	0.61	[0.58, 0.64]
*I* parameter	0.53	[0.50, 0.57]	0.59	[0.55, 0.63]	0.53	[0.50, 0.57]	0.59	[0.55, 0.62]

*Note.* Full sample refers to the data before exclusion of response-time outliers; reduced sample refers to the data after exclusion of response-time outliers. Moral-dilemma indices scores can range from 0 to 12. CNI model parameter scores can range from 0 to 1. CI = confidence interval.

#### Exploratory Response-Time Analysis

To explore whether the obtained effects on moral judgments are related to differences in the resources participants devoted to thinking about their responses, we again created an index of response time by calculating the total time participants spent on the moral dilemmas in the battery. Exploratory analyses revealed that response times did not significantly differ across groups (*M*_think-about-reasons_ = 1436.68; *M*_rely-on-intuitions_ = 1375.38), *t*(247) = −1.23, *p* = .220, *d* = 0.156. To rule out undue effects of response-time outliers, we computed the median absolute deviation (*MAD* = 377.23) for total response time and applied a moderately conservative criterion in which cases are identified as outliers if they lie beyond 2.5 absolute deviations from the median (Leys et al., 2013). Using this procedure, six cases were flagged as potential outliers (*n*_rely-on-intuitions_ = 2; *n*_think-about-reasons_ = 4). After excluding these outliers, response times still did not significantly differ across conditions (*M*_think-about-reasons_ = 1395.28; *M*_rely-on-intuitions_ = 1358.56), *t*(241) = 0.02, *p* = .981, *d* = 0.003. The CNI model fit the data well with the reduced sample, *G*^2^(2) = 0.23, *p* = .891, *w* = 0.004. Consistent with the results obtained with the full sample, analyses on the reduced sample yielded a significant group difference in the *C* parameter, Δ ∆*G*^2^(1) = 4.12, *p* = .042, *d* = 0.262, *N* parameter, Δ ∆*G*^2^(1) = 6.07, *p* = .014, *d* = 0.318, and *I* parameter, Δ ∆*G*^2^(1) = 3.88, *p* = .049, *d* = 0.255.

### Discussion

Replicating the effect on the *N* parameter in Experiment 1, thinking about reasons increased sensitivity to moral norms in moral-dilemma judgments. However, inconsistent with the null effect on the *C* parameter in Experiment 1, thinking about reasons also increased sensitivity to consequences. The *I* parameter also showed inconsistent effects across studies, in that thinking about reasons increased general preference for *action* in Experiment 1 and general preference for *inaction* in Experiment 2. Supporting the idea that thinking about reasons influences moral-dilemma judgments independent of the amount of invested resources, response times in the two conditions did not significantly differ from each other.

## Experiment 3

One question that the prior two experiments did not address pertains to whether the identified effects on the *N* parameter are driven specifically by the act of thinking about reasons for one’s choices or by the act of thinking in general, irrespective of the thought content. Experiment 3 aimed to address this question. Toward this end, Experiment 3 procedurally mirrored the prior two studies, the main difference being that it included an additional condition in which participants were instructed to think about their intuitions rather than reasons that justify their choices. We sought to replicate the findings of Experiments 1 and 2, and further tested the hypothesis that participants who are prompted to think about reasons will be more sensitive to moral norms than those who are prompted to think about their intuitions and those who are prompted to rely on their intuitions. The study was formally preregistered at https://osf.io/rg6ew/.

### Method

#### Participants

Participants were recruited via Prolific Academic. To be eligible for the study, participants had to (a) be at least 18 years old, (b) be fluent in English, (c) have the United Kingdom as their registered home country, (d) have a minimum approval rate of 95% on past assignments, (e) have completed at least 100 studies on Prolific, and (f) have not participated in studies from the authors’ lab that used the same moral-dilemma battery. We preregistered to recruit 600 participants for Experiment 3. Sensitivity analyses conducted using G*Power 3.1 (Faul et al., 2007) suggested that a sample of *N* = 510 (*n* = 170) can detect effect sizes of *f* = .14 with 80% power and α = .05 (two-tailed). Because we expected that approximately 15% of the sample would fail the attention check, we oversampled and recruited 600 participants (*n* = 200 per condition) for the study. Participants were compensated $6.00.

We received a total of 614 submissions, of which 601 were complete submissions; one additional participant completed the study but was initially excluded from Prolific’s list of complete submissions due to a technical error. Of the 601 complete submissions, 16.3% failed the attention check. The final sample size thus included 503 participants (*n*_rely-on-intuitions_ = 164; *n*_think-about-intuitions_ = 174; *n*_think-about-reasons_ = 165). Participant demographics are as follows: 57.3% female, 41.2 % male, 1.6% other/I prefer not to answer; *M*_age_ = 39.3 years; 89.9% White, 7.0% Asian, 3.2% Black, 0.6% Hispanic, Latino, or Spanish origin, 0.6% Middle Eastern, and 0.6% other ethnicities.

#### Procedure and Measures

The procedure of Experiment 3 largely followed that of Experiment 1 with three exceptions. First, Experiment 3 included three rather than two experimental conditions: (a) rely on intuitions, (b) think about reasons, and (c) think about intuitions. Similar to the intructions in Experiments 1 and 2, participants in the *rely-on-intuitions* condition read the following instructions: *For each story, we ask that you trust your first intuitive and spontaneous “gut” reactions and respond accordingly. You do not have to explain your intuitions.* To reiterate this instruction, we placed the statement *When you make your judgment, you should trust your first intuitive and spontaneous “gut” reactions* above each moral-dilemma question. Also similar to the instructions in Experiments 1 and 2, participants in the *think-about-reasons* condition received the following instructions: *For each story, we ask that you take a moment to think about reasons that would justify your responses and respond accordingly. At the end of the study, you will be asked to explain your reasoning.* Participants in this condition were given the following reminder which was placed above each moral-dilemma question: *When you make your judgment, you should take a moment to think about reasons that justify your responses.* Finally, participants in the newly included *think-about-intuitions* condition read the following instructions: *For each story, we ask that you take a moment to think about your intuitive and spontaneous “gut” reactions and respond accordingly. You do not have to explain your intuitions.* Participants in this condition read the following reminder that was placed above each moral-dilemma question: *When you make your judgment, you should take a moment to think about your intuitive and spontaneous “gut” reactions.*

Another procedural difference between Experiments 3 and 1 is the number of attention checks that participants had to pass. Experiment 3 included only one reading-intensive attention-check item, which instructed participants to not select any options but instead skip ahead to the next screen. Participants who selected any of the response options were thus considered to have failed the attention check. Finally, we reduced the number of demographic questions posed to participants in Experiment 3, such that participants only reported their gender, age, and ethnicity.

#### Data Analysis

With three experimental conditions, the CNI model has a total of 12 free categories (i.e., four types of dilemmas for each of the three conditions) and nine parameters (i.e., three parameters estimated for each of the three conditions), resulting in three degrees of freedom. We preregistered that we will first fit the data to a baseline model that freely estimates the three CNI model parameters within each of the three experimental conditions. To test the hypothesis that there will be a significant difference between conditions on the *N* parameter, we preregistered that we will fit a model in which the *N* parameter is constrained to be equivalent across conditions, and then compare the fit of the constrained model to the fit of the baseline model. For the next step, we preregistered that we will constrain the *N* parameter to be equal across each pair of conditions and compare each model’s fit to that of the baseline model to identify which pairs of conditions have significantly different estimates of the *N* parameter. For comprehensiveness, we conducted the same set of analyses for the *C* and *I* parameters.

### Results

#### Exploratory Traditional Dilemma Analysis

Responses to the traditional dilemma variant were marginally different across conditions, *F*(2, 500) = 2.77, *p* = .063, $\eta_{p}^{2}$ = .011. Post hoc tests with Bonferroni correction revealed that the largest difference in the traditional score was between the think-about-reasons condition (*M* = 4.20, 95% CI [3.83, 4.57]) and the think-about-intuitions condition (*M* = 4.78, 95% CI [4.42, 5.14]), *p* = .082, 95% CI [-.05, 1.21], with the rely-on-intuitions condition falling between the two thinking conditions (*M* = 4.68, 95% CI [4.31, 5.05]).

#### Preregistered CNI Model Analysis

The CNI model fit the data well, *G*^2^(3) = 1.55, *p* = .670, *w* = 0.008. Means and 95% confidence intervals of the estimated parameter scores are presented in Table 3. Preregistered confirmatory analyses revealed that the *N* parameter was significantly different across conditions, Δ ∆*G*^2^(2) = 19.72, *p* < .001, *w* = 0.029, indicating that participants in the think-about-reasons condition were significantly more sensitive to moral norms than those in the rely-on-intuitions condition, Δ ∆*G*^2^(1) = 10.74, *p* = .001, *d* = 0.363, and those in the think-about-intuitions condition, Δ ∆*G*^2^(1) = 17.98, *p* < .001, *d* = 0.462. Participants in the rely-on-intuitions condition and think-about-intuitions condition did not significantly differ in terms of sensitivity to moral norms, Δ ∆*G*^2^(1) = 0.83, *p* = .364, *d* = 0.099. Sensitivity to consequences did not significantly differ across the three conditions, Δ ∆*G*^2^(2) = 1.75, *p* = .418, *w* = 0.009. Likewise, we did not find any significant differences across conditions on the *I* parameter, Δ ∆*G*^2^(2) = 2.39, *p* = .302, *w* = 0.010.

**Table 3.** Means and 95% Confidence Intervals of Moral Dilemma Action (vs. Inaction) Indices, and CNI Model Parameters, Experiment 3.

	Full sample (*N* = 503)						Reduced sample (*N* = 475)					
	Rely on intuitions (*n* = 164)		Think about intuitions (*n* = 174)		Think about reasons (*n* = 165)		Reply on intuitions (*n* = 156)		Think about intuitions (*n* = 163)		Think about reasons (*n* = 156)	
	*M*	95% CI	*M*	95% CI	*M*	95% CI	*M*	95% CI	*M*	95% CI	*M*	95% CI
Moral-dilemma indices												
Proscriptive norm prohibits action												
Benefits of action > costs	4.68	[4.33, 5.03]	4.78	[4.41, 5.15]	4.20	[3.82, 4.58]	4.67	[4.31, 5.02]	4.75	[4.37, 5.13]	4.19	[3.79, 4.59]
Benefits of action < costs	1.57	[1.33, 1.80]	1.52	[1.27, 1.78]	1.25	[1.04, 1.47]	1.51	[1.28, 1.75]	1.50	[1.24, 1.77]	1.22	[1.01, 1.44]
Prescriptive norm prescribes action												
Benefits of action > costs	10.13	[9.88, 10.39]	9.95	[9.68, 10.23]	10.28	[10.06, 10.51]	10.16	[9.91, 10.42]	9.98	[9.70, 10.27]	10.29	[10.05, 10.52]
Benefits of action < costs	6.84	[6.50, 7.17]	6.73	[6.36, 7.10]	7.12	[6.78, 7.47]	6.85	[6.50, 7.19]	6.77	[6.40, 7.14]	7.13	[6.77, 7.49]
CNI model parameters												
*C* parameter	0.27	[0.25, 0.29]	0.27	[0.25, 0.29]	0.25	[0.24, 0.27]	0.27	[0.25, 0.29]	0.27	[0.25, 0.29]	0.25	[0.24, 0.27]
*N* parameter	0.61	[0.58, 0.63]	0.59	[0.57, 0.62]	0.67	[0.64, 0.69]	0.62	[0.59, 0.64]	0.60	[0.57, 0.62]	0.67	[0.65, 0.70]
*I* parameter	0.55	[0.52, 0.58]	0.57	[0.54, 0.60]	0.59	[0.55, 0.62]	0.56	[0.52, 0.59]	0.57	[0.54, 0.60]	0.59	[0.56, 0.63]

*Note.* Full sample refers to the data before exclusion of response-time outliers; reduced sample refers to the data after exclusion of response-time outliers. Moral-dilemma indices scores can range from 0 to 12. CNI model parameter scores can range from 0 to 1. CI = confidence interval.

#### Exploratory Response-Time Analysis

To verify that the identified experimental effect on the *N* parameter is not attributable to deliberation duration rather than thought content, we again created an index of response time by calculating the total time participants spent on the moral dilemmas. Exploratory analyses revealed that response times did not significantly differ across conditions (*M*_rely-on-intuitions_ = 1291.67; *M*_think-about-intuitions_ = 1346.04; *M*_think-about-reasons_ = 1336.51), *F*(2, 500) = 0.45, *p* = .641, $\eta_{p}^{2}$ = .002. To rule out undue effects of response-time outliers, we again computed the median absolute deviation (*MAD* = 400.29) for total response time and applied a moderately conservative criterion in which cases are identified as outliers if they lie beyond 2.5 absolute deviations from the median (Leys et al., 2013). Using this procedure, 28 cases were flagged as potential outliers (*n*_rely-on-intuitions_ = 8; *n*_think-about-intuitions_ = 11; *n*_think-about-reasons_ = 9). After excluding these outliers, response times still did not significantly differ across conditions (*M*_rely-on-intuitions_ = 1208.90; *M*_think-about-intuitions_ = 1237.39; *M*_think-about-reasons_ = 1234.88), *F*(2, 472) = 0.27, *p* = .760, $\eta_{p}^{2}$ = .001. The CNI model fit the data well with the reduced sample, *G*^2^(3) = 1.19, *p* = .755, *w* = 0.007. Consistent with the results obtained with the full sample, analyses on the reduced sample revealed a significant difference in the *N* parameter across conditions, Δ ∆*G*^2^(2) = 17.01, *p* < .001, *w =* 0.027, such that participants who thought about reasons were significantly more sensitive to moral norms than those who responded intuitively, Δ *G*^2^(1) = 8.68, *p* = .003, *d* = 0.335, and those who thought about their intuitions, Δ ∆*G*^2^(1) = 15.83, *p* < .001, *d* = 0.447. Participants in the rely-on-intuitions condition and think-about-intuitions condition did not significantly differ in their sensitivity to moral norms, Δ ∆*G*^2^(1) = 0.98, *p* = .322, *d* = 0.111. Also consistent with the full-sample analyses, no significant differences were found for the *C* parameter, Δ ∆*G*^2^(2) = 1.48, *p* = .477, *w =* 0.008, and *I* parameter, Δ ∆*G*^2^(2) = 2.23, *p* = .328, *w =* 0.010, in the reduced-sample analyses.

### Discussion

The findings of Experiment 3 suggest that the effect on norm sensitivity identified in Experiments 1 and 2 is driven specifically by the act of thinking about reasons for one’s choices rather than the act of thinking in general, irrespective of thought content. Consistent with this conclusion, participants who were prompted to think about reasons showed a stronger sensitivity to moral norms compared with both (a) participants who were promoted to rely on their intuitions and (b) participants who were promoted to think about intuitions. Because response times did not significantly differ across conditions, the findings of Experiment 3 also provide further support for the idea that thinking about reasons influences moral judgments independent of the amount of invested resources.

## Integrative Data Analysis

To address potential issues of statistical underpowering, we conducted an integrative data analysis (IDA; Curran & Hussong, 2009) comparing the responses of participants who were instructed to think about reasons for their choices (i.e., *think-about-reasons* condition; *N* = 368) with responses of participants not instructed to think about reasons (i.e., *no-reasons* condition; *N* = 549), with Experiment 3’s rely-on-intuitions and think-about-intuitions conditions merged into the latter. Because the larger sample size in this IDA should reduce the likelihood of both false negatives (Maxwell et al., 2015) and false positives (Button et al., 2013), it permits stronger conclusions regarding how thinking about reasons influences moral-dilemma judgments.

### Traditional Dilemma Analysis

We did not find any difference in relative preference for utilitarian over deontological judgments across the think-about-reasons and no-reasons conditions, *t*(915) = 1.31, *p* = .191, *d* = 0.088.

### CNI Model Analysis

Standardized response times did not significantly differ across conditions in the pooled dataset (*M*_think-about-reasons_ = 0.07; *M*_no-reasons_ = −0.05), *t*(692.30) = −1.67, *p* = .095, *d* = 0.117. Forty-eight cases laid beyond 2.5 absolute deviations from the median and were thus flagged as potential outliers (*n*_no-reasons_ = 23; *n*_think-about-reasons_ = 25). Standardized response times still did not significantly differ across conditions when the 48 potential outliers were excluded (*M*_think-about-reasons_ = −0.13; *M*_no-reasons_ = −0.17), *t*(867) = −0.85, *p* = .396, *d* = 0.059. The CNI model fit the data well for both the full and reduced pooled datasets, *G*^2^(2)s ≤ 1.32, *p* ≥ .517, *w*s ≤ 0.005. Means and 95% confidence intervals of the estimated parameter scores are presented in Table 4. The IDA revealed a significant difference on the *N* parameter across conditions regardless of whether response-time outliers were removed, Δ ∆*G*^2^(1) > 16.81, *p* < .001, *d*_full_ = 0.310, *d*_reduced_ = 0.284. We did not find any significant differences on the *C* parameter, Δ ∆*G*^2^(1)s < 0.04, *p*s > .837, *d*_full_ = 0.014, *d*_reduced_ = 0.006, and *I* parameter, Δ *G*^2^(1)s < 0.43, *p*s > .514, *d*_full_ = 0.023, *d*_reduced_ = 0.046, in both the full and reduced pooled samples.

**Table 4.** Means and 95% Confidence Intervals of Moral Dilemma Action (vs. Inaction) Indices, and CNI Model Parameters, Pooled Data From Experiments 1 to 3.

	Full sample (*N =* 917)				Reduced sample (*N =* 869)			
	No reasons (*n* = 549)		Think about reasons (*n* = 368)		No reasons (*n* = 526)		Think about reasons (*n* = 343)	
	*M*	95% CI	*M*	95% CI	*M*	95% CI	*M*	95% CI
Moral-dilemma indices								
Proscriptive norm prohibits action								
Benefits of action > costs	4.93	[4.73, 5.14]	4.71	[4.43, 4.98]	4.93	[4.72, 5.14]	4.73	[4.45, 5.02]
Benefits of action < costs	1.83	[1.67, 1.99]	1.60	[1.38, 1.81]	1.83	[1.66, 1.99]	1.59	[1.36, 1.81]
Prescriptive norm prescribes action								
Benefits of action > costs	9.93	[9.79, 10.08]	10.15	[9.99, 10.31]	9.95	[9.80, 10.10]	10.13	[9.96, 10.30]
Benefits of action < costs	6.70	[6.50, 6.90]	6.96	[6.72, 7.21]	6.72	[6.52, 6.92]	6.93	[6.67, 7.19]
CNI model parameters								
*C* parameter	0.26	[0.25, 0.27]	0.26	[0.25, 0.27]	0.26	[0.25, 0.27]	0.26	[0.25, 0.28]
*N* parameter	0.56	[0.54, 0.57]	0.61	[0.59, 0.63]	0.56	[0.54, 0.58]	0.61	[0.59, 0.63]
*I* parameter	0.54	[0.52, 0.55]	0.54	[0.52, 0.56]	0.53	[0.52, 0.55]	0.54	[0.52, 0.56]

*Note.* Full sample refers to the pooled data before exclusion of response-time outliers; reduced sample refers to the pooled data after exclusion of response-time outliers. CNI model parameter scores can range from 0 to 1. CI = confidence interval.

## General Discussion

Using the CNI model to disentangle sensitivity to consequences, sensitivity to moral norms, and general preference for inaction versus action (Gawronski et al., 2017), the current research investigated how thinking about reasons for one’s choices influences moral-dilemma judgments. Consistent with the mixed and inconclusive effects of cognitive reflection on moral judgments in the trolley problem, thinking about reasons had no discernable effect on participants’ relative preference for utilitarian over deontological judgments in dilemmas that are structurally similar to the trolley problem (i.e., wherein the action is proscribed by moral norms of harm and brings about benefits to the greater good). However, because relative preference for utilitarian over deontological judgments is a “noisy” measure with at least three conceptually distinct sources of variance (see Gawronski et al., 2020), the obtained null effect may conceal reliable effects on any one of the three underlying factors. Indeed, when the confounds in the traditional dilemma paradigm were resolved by means of the CNI model, thinking about reasons for one’s choices reliably increased sensitivity to moral norms in moral-dilemma judgments. This effect replicated across three studies (two preregistered) with MTurk workers (Experiment 1), undergraduate students (Experiment 2), and Prolific workers (Experiment 3) and with samples from the United States (Experiments 1 and 2) and the United Kingdom (Experiment 3). Although thinking about reasons for one’s choices significantly increased sensitivity to consequences in Experiment 2, no such effect was obtained in Experiments 1 and 3 as well as the IDA. Results for general action preferences were similarly inconsistent, in that thinking about reasons increased general preference for action in Experiment 1 and general preference for inaction in Experiment 2. There was no significant effect on general action preferences in Experiment 3 and the IDA. Thus, the only robust, reproducible effect identified in the current set of experiments is the finding that thinking about reasons for one’s choices increased sensitivity to moral norms.

To avoid potential misinterpretations, it is worth clarifying what the current findings do and do not suggest. Critically, the current findings do *not* suggest that participants in the think-about-reasons condition had thought more about norm-based reasons rather than consequence-based reasons. Participants who were prompted to think about reasons could have generated various kinds of reasons to justify their responses, but the generated reasons do not have to be rooted in deontological norms of harm. Hence, the current research does not demonstrate that prompting participants to think about reasons caused them to think more about specific deontic norms. Instead, the current research demonstrates that thinking about reasons increased sensitivity to moral norms in a manner such that participants became more likely to object to actions that cause proximal harm and support actions that prevent proximal harm, and that this is irrespective of the specific reasons participants thought about.

Importantly, our results further suggest that thinking about reasons increased sensitivity to moral norms independent of the amount of time participants spent thinking about their responses. Although participants in the think-about-reasons condition spent more time deliberating than participants in the intuition condition in Experiment 1, no such difference was found in Experiments 2 and 3 as well as the IDA. Moreover, any identified differences in response times disappeared when response-time outliers were excluded from analyses. Yet, the obtained effect of thinking about reasons on moral judgments remained reliable, in that thinking about reasons increased sensitivity to moral norms in the three experiments and the IDA, regardless of whether response-time outliers were excluded. Together, these results are consistent with the idea that thinking about reasons increased sensitivity to moral norms via the contents of participants’ thought processes (intuitions versus reasons) instead of the amount of invested resources (high versus low elaboration).

Experiment 3 further demonstrated that the identified effect on sensitivity to moral norms was driven specifically by the act of thinking about reasons rather than the act of thinking in general. There is a clear difference between thinking *that* X is acceptable (or unacceptable) and thinking about reasons *why* X is acceptable (or unacceptable). If the mere act of thinking is what drove the increase in sensitivity to moral norms, Experiment 3 should have revealed (a) a significant difference on the *N* parameter between the think-about-intuitions condition and the rely-on-intuitions condition, and (b) no significant difference on the *N* parameter between the think-about-intuitions condition and the think-about-reasons condition, because the content of one’s thoughts should be irrelevant. Different from these predictions, we found (a) a significant difference on the *N* parameter between the think-about-reasons condition and the think-about-intuitions condition, and (b) no significant difference on the *N* parameter between the think-about-intuitions condition and the rely-on-intuitions condition. These results suggest that thinking about reasons, not thinking in general, increased sensitivity to moral norms.

### Implications

The current findings have important implications for extant theories about the processes underlying moral judgments. Some theories suggest that the reasons people generate for their moral judgments are mere post hoc rationalizations of moral intuitions whose origins are outside of awareness (Haidt, 2001). According to this view, moral judgments should not differ depending on whether participants are asked to think about reasons or focus on intuitions, because thoughts about reasons are not causally involved in producing moral judgments; they are mere afterthoughts to moral intuitions arising from unconscious processes. Although it is possible that moral judgments are sometimes rooted in moral intuitions whose origins are outside of awareness, the current findings demonstrate that generated reasons can play a causal role in moral judgments instead of always being inconsequential afterthoughts to moral intuitions.

Contrary to intuitionist accounts’ proposition about the causal ineffectiveness of generated reasons (Haidt, 2001), the DPM implies the possibility that, although the reasons generated for deontological judgments may be mere post hoc rationalizations of unconsciously generated intuitions, thinking about reasons may enhance utilitarian judgments by promoting cost–benefit analyses (Greene, 2007). According to this view, thinking about reasons for one’s choices may influence sensitivity to consequences without affecting sensitivity moral norms. The current studies obtained the opposite pattern, in that thinking about reasons for one’s choices increased sensitivity to moral norms without affecting sensitivity to consequences. These results suggest that norm-conforming judgments in moral dilemmas can arise from reflective thoughts about reasons, challenging the ideas that norm-conforming judgments in moral dilemmas are the exclusive product of automatic emotional responses and that reflective processes influence moral-dilemma judgments by overriding the impact of norm-conforming intuitions.

Why might thinking about reasons increase sensitivity to moral norms? As mentioned, moral reasoning need not simply involve considerations of the costs and benefits associated with an action but can also involve deeper reflections of deontological principles (e.g., considerations of the ripple effects of violating deontological norms). Another potential answer to this question could be derived from the interpersonal nature of reasons and moral judgments. Reasons are expected to be socially convincing, with failure to persuasively justify one’s beliefs or behaviors often linked to negative social consequences (Lerner & Tetlock, 1999; Wilson et al., 1989). Judgments of morality are also not purely intrapersonal phenomena formed in a social vacuum (Plaks et al., 2022; Robinson et al., 2015); instead, moral judgments may inherently involve considerations of how one might successfully justify one’s choices to real or imagined interlocutors (Scanlon, 1998). Demonstrating the interpersonal nature of moral judgments, recent work has shown that concerns about being negatively evaluated by others are associated with weaker relative preference for utilitarian over deontological judgments (Hashimoto et al., 2022). Because norm-conforming judgments are perceived more favorably than outcome-maximizing judgments (Everett et al., 2016; Rom & Conway, 2018; Turpin et al., 2021), and work using the CNI model indicates that these perceptions are linked to people’s sensitivity to moral norms rather their sensitivity to consequences or general action preferences (Gawronski, 2022), thinking about reasons for one’s choices may shift moral-dilemma judgments toward socially approved options, which tend to be sensitive to moral norms. Research illuminating whether thinking about reasons increases sensitivity to moral norms through greater deontological reasoning, heightened social considerations, or both is needed.

The current findings also highlight the importance of distinguishing between *degree* and *content* in research on cognitive reflection. Whereas the former refers to the mental resources that are available for or invested in processing judgment-relevant information, the latter refers to the content of judgment-relevant information. By manipulating cognitive load and time pressure, prior research obtained mixed results on how different degrees of cognitive reflection influence moral-dilemma judgments (i.e., high versus low elaboration), but such manipulations remain silent about the contents of the underlying thought processes (i.e., thoughts about intuitions versus reasons). The current research goes beyond this work by showing that thinking about reasons influences moral-dilemma judgments independent of the invested amount of mental resources. Future research may integrate the two approaches via independent manipulations of the degree and content of cognitive reflection.

### Limitations and Supplemental Analyses

One limitation of the current experiments is the lack of a manipulation check, which would have ideally taken the form of a measure of participants’ thought contents. While including such a measure would have bolstered the current research by allowing us to confirm if participants’ thought contents did indeed differ across conditions, accessing participants’ thought contents is a challenging task when they are asked to rely on or think about their intuitions. If we had asked participants in these conditions to express their thoughts during the moral-dilemma task, we would have risked priming participants to think about reasons for their judgments. If we had asked participants in these conditions to express their thoughts after the moral-dilemma task, the expressed thoughts may reflect mere post hoc rationalizations of participants’ prior choices (see Stanley et al., 2018). In either case, differences in thought contents across experimental conditions would remain uninformative about the intended difference between thinking about reasons versus intuitions.

Although the CNI model is superior to the traditional trolley paradigm for its ability to disentangle three distinct factors underlying moral-dilemma judgments, the model also has some limitations (Baron & Goodwin, 2020, 2021; Liu & Liao, 2021). Anticipating concerns about the potential impact of these limitations on our findings, we conducted three sets of supplemental analyses on the pooled data to (a) address potential concerns about specific items of the moral-dilemma battery and (b) test the robustness of the identified effect of thinking about reasons on the *N* parameter. First, we conducted follow-up analyses to determine whether the results differ after excluding the four variants of five potentially problematic dilemmas (i.e., using only responses to seven scenarios in their four variants). One dilemma had previously been found to have low construct validity in the manipulation of moral norms (see Gawronski et al., 2020); four additional dilemmas confound the norm manipulation with whether the focal action involves interference with the action of someone else. Although the obtained effect sizes were much smaller after excluding the four variants of the five potentially problematic dilemmas, the results revealed the same effect of thinking about reasons on sensitivity to moral norms (Table S1 of Supplemental Materials).

Next, we conducted two sets of supplemental analyses to test the robustness of the identified effects against the assumed hierarchical structure of the CNI model parameters in the processing tree (see Figure 1). Although there are methodological reasons to include the *I* parameter at the lowest level in the processing tree (see Gawronski et al., 2017, 2020),^4^ the position of the *C* and *N* parameters are arbitrary in the sense that a model in which the two parameters are included in reverse order (i.e., NCI) yields the same goodness-of-fit as the original CNI model. Nevertheless, estimates for *N* and *C* are different across the two models, because the parameter at the lower level is estimated conditional upon the parameter at the higher level, which could lead to different relations to the same external variable (e.g., thinking about reasons). To address this concern, we conducted follow-up analyses to determine whether the current results differ when the data are analyzed with a model in which the positions of the *C* and *N* parameters are reversed. We also conducted supplemental analyses using an alternative procedure called the CAN algorithm, which algebraically calculates the three model parameters concurrently rather than hierarchically (see Liu & Liao, 2021). Although the obtained effect sizes in the analyses with the CAN algorithm were much smaller compared with the ones with the CNI model, thinking about reasons had the same effect on sensitivity to moral norms in the analyses using the CAN algorithm and the NCI model (Tables S2 to S4 of Supplemental Materials).

## Conclusion

Thinking about reasons can influence how we evaluate choice options. In the context of moral-dilemma judgments, the current findings indicate that thinking about reasons for one’s choices increases sensitivity to moral norms in responses to moral dilemmas. This finding conflicts with the prevailing assumptions that norm-conforming judgments in moral dilemmas are the exclusive product of automatic emotional responses and that the primary function of reflective reasoning is to override the effects of automatic emotional responses.

## Supplemental Material

sj-docx-1-psp-10.1177_01461672231180760 – Supplemental material for Thinking About Reasons for One’s Choices Increases Sensitivity to Moral Norms in Moral-Dilemma Judgments Supplemental material, sj-docx-1-psp-10.1177_01461672231180760 for Thinking About Reasons for One’s Choices Increases Sensitivity to Moral Norms in Moral-Dilemma Judgments by Nyx L. Ng, Dillon M. Luke and Bertram Gawronski in Personality and Social Psychology Bulletin

## Notes

1. The discrepancy between the effect size reported in Experiment 1 and the effect size utilized for the power analysis in the preregistration in Experiment 2 is due to a data-cleaning error in our initial analyses. Four participants who completed the moral-dilemma battery but not the remaining sections of the study were mistakenly retained in the initial analyses. Likewise, one participant who failed the second attention check was mistakenly retained in the initial analyses. After correcting for these errors, the effect size for the obtained difference on the *N* parameter increased from *d* = 0.368 to *d* = 0.535. With an assumed effect size of *d* = 0.535, a sample of 250 participants provides a power of 99% for the detection of a difference between two independent means using an alpha criterion of .05 (two-tailed).

2. Due to an administrative error by the research assistants who ran the study, three participants who were supposed to be assigned to the think-about-reasons condition were mistakenly assigned to the rely-on-intuitions condition.

3. When we included the seven participants who completed the study after we had reached our preregistered target sample (*n*_rely-on-intuitions_ = 132; *n*_think-about-reasons_ = 124), the analyses largely produced the same results, such that there was a statistically significant difference between conditions for the *C* parameter, Δ ∆*G*^2^(1) = 4.46, *p* = .035, *d* = 0.265, and the *N* parameter Δ ∆*G*^2^(1) = 5.70, *p* = .017, *d* = 0.300. However, the significant difference obtained for the *I* parameter in the preregistered analysis was only marginal when the seven additional participants were included, Δ ∆*G*^2^(1) = 3.66, *p* = .056, *d* = 0.224.

4. Within multinomial modeling, the response patterns predicted for the absence of all specified factors are defined as the opposite pattern of the parameter at the lowest position in the hierarchy (i.e., general action as the opposite of general inaction). Thus, setting the *I* parameter as the lowest parameter in the processing tree permits an estimation of general action preferences along a bipolar continuum of inaction versus action (rather than a unipolar dimension of general preference for inaction).
