# Supplementary material for: Thinking About Reasons for One’s Choices Increases Sensitivity to Moral Norms in Moral-Dilemma Judgments
Source: Pers Soc Psychol Bull. 2023 Jun 29;51(1):33–48. doi: 10.1177/01461672231180760 (PMC11616218; doi:10.1177/01461672231180760)
Supplement: sj-docx-1-psp-10.1177_01461672231180760 – Supplemental material for Thinking About Reasons for One’s Choices Increases Sensitivity to Moral Norms in Moral-Dilemma Judgments [file sj-docx-1-psp-10.1177_01461672231180760.docx]

**Supplemental Materials:**

**Thinking about Reasons for One’s Choices Increases Sensitivity to Moral Norms in Moral-Dilemma Judgments**

# Mixed Model Analysis of Four Dilemma Variants

For the sake of comprehensiveness, we also analyzed the effects of thinking about reasons on responses to all four dilemma variants and not just the traditional variant (i.e., where the focal norm prohibits an action that brings about greater benefits than costs). Using the combined data from all three studies, the four action indices were submitted to a 2 (Norm: proscriptive vs. prescriptive) × 2 (Consequence: greater vs. smaller) × 2 (Condition: think-about-reasons vs. no-reasons) mixed ANOVA with the first two variables as within-subjects factors and the third as a between-subjects factor. The results indicate a significant main effect of the norm manipulation, *F*(1, 915) = 2423.90, *p* < .001, $\eta_{p}^{2}$ = .726, such that participants were more likely to judge an action as acceptable in the dilemmas when the action was prescribed (*M* = 8.44, *SE* = 0.06) rather than prohibited (*M* = 3.27, *SE* = 0.07) by a norm. This effect was moderated by whether participants thought about reasons for their responses or were not prompted to do so, *F*(1, 915) = 5.05, *p* = .025, $\eta_{p}^{2}$ = .005, with the gap between judgments of acceptability for proscriptive versus prescriptive dilemmas being greater in the think-about-reasons condition (*M*_difference_ = 5.41, *SE* = .16), *F*(1, 915) = 1106.64, *p* < .001, $\eta_{p}^{2}$ = .547, than in the no-reasons condition (*M*_difference_ = 4.93, *SE* = .13), *F*(1, 915) = 1375.34, *p* < .001, $\eta_{p}^{2}$ = .600. A significant main effect of consequences also emerged, *F*(1, 915) = 5.05, *p* < .001, $\eta_{p}^{2}$ = .670, indicating that participants were more likely to judge an action as acceptable if it brings about greater benefits than costs (*M* = 7.43, *SE* = 0.04) as compared to when it brings about more costs than benefits (*M* = 4.27, *SE* = 0.05). This effect was not qualified by the norm manipulation and the reasons manipulation, and the three-way interaction was also non-significant, *F*s < 2.26, *p*s > .133, $\eta_{p}^{2}s$ < .002. Together, the results corroborate our conclusion that, when forming judgments of moral acceptability, thinking about reasons influences sensitivity to moral norms but not sensitivity to consequences.

# Robustness against Dilemma Exclusion

An item-based analysis by Gawronski et al. (2020) revealed that one of the 12 dilemmas used in the current research has low construct validity in the manipulation of moral norms (*abduction dilemma*). Moreover, four of the remaining 11 dilemmas can be criticized for confounding moral norms with whether the focal action requires interference with the action of someone else (*transplant dilemma, torture dilemma, vaccine dilemma,* and *tyrant-killing dilemma*). Using the pooled data, we conducted supplemental analyses with these five dilemmas excluded.

Response times to the four variants of the remaining seven dilemmas (i.e., 28 dilemmas in total) were summed and re-standardized for each individual experiment. Forty-eight cases were flagged as potential outliers based on the standardized response times to the dilemma included in the current analysis (*n*_no-reasons_ = 27; *n*_think-about-reasons_ = 21). Response times did not differ across the think-about-reasons and no-reasons conditions regardless of whether potential outliers were excluded, *t*s > -0.82, *p*s > .413, *d_full_* = 0.055, *d_reduced_* = 0.041. The CNI model did not fit the pooled data well regardless of whether the full or reduced sample were analyzed, *G^2^*(2)s > 28.87, *p*s <.001, *w*s ≥ 0.034. Nevertheless, a significant between-condition difference in the *N* parameter emerged, $\Delta$*G^2^*s > 4.54, *p*s < .033, *d_full_* = 0.144, *d_reduced_* = 0.166, consistent with the results obtained with the full dilemma set. There were no significant group differences in the *C* parameter, $\Delta$*G^2^*s < 0.16, *p*s ≥ .692, *d_full_* = 0.021, *d_reduced_* = 0.026, and the *I* parameter, $\Delta$*G^2^*s < 1.03, *p*s ≥ .310, *d_full_* = 0.043, *d_reduced_* = 0.072. Means and 95% confidence intervals of the three model parameters after exclusion of the potentially problematic dilemmas are presented in Table S1.

# Robustness against Model Assumptions

Another potential issue with the CNI model pertains to its hierarchical structure. Given the arbitrary positions of the *C* and *N* parameters in the CNI model processing tree, we re-analyzed the pooled data using an alternative model in which the positions of the *C* and *N* parameters are reversed (for the sake of brevity called NCI model). We also re-analyzed the pooled data using the CAN algorithm, which algebraically calculates the three model parameters concurrently rather than hierarchically (Liu & Liao, 2021).

### Re-analyses using the NCI Model

Means and 95% confidence intervals of the three parameters obtained with the NCI model are presented in Table S2. The NCI model fit the pooled data well, *G^2^*(2)s ≤ 1.32, *p*s ≥ .517, *w*s < 0.01, regardless of whether response-time outliers were removed. Consistent with the CNI model analyses, significant between-group differences were found for sensitivity to moral norms regardless of whether potential response-time outliers were removed, $\Delta$*G^2^*s > 16.16, *p*s < .001, *d_full_* = 0.316, *d_reduced_* = 0.279. The NCI model analysis revealed no significant differences in general preference for inaction versus action both before and after outlier exclusion, $\Delta$*G^2^*s < 0.43, *p*s > .514, *d_full_* = 0.023, *d_reduced_* = 0.046. Contrary to the integrative CNI model analyses, the NCI model analyses revealed a significant difference in sensitivity to consequences across conditions, $\Delta$*G^2^*s ≥ 4.05, *p*s < .044, *d_full_* = 0.138, *d_reduced_* = 0.141, suggesting that participants in the think-about-reasons condition were more sensitive to consequences than those who responded intuitively or thought about their intuitions.

### Re-analyses using the CAN Algorithm

Means and 95% confidence intervals of the model parameters obtained using the CAN algorithm are presented in Table S3. Because the parameters of the CAN algorithm tend to be highly correlated (which is not the case for the CNI model parameters), it is important to control for shared variances in analyses using the CAN algorithm to avoid false positive results for a given parameter. We therefore conducted analyses of covariance to test effects of our experimental manipulation on each of three CAN algorithm parameters while entering the remaining two parameters as covariates (Table S4). The ANCOVA predicting the *N* parameter revealed a significant experimental main effect, *F*s > 4.31, *p*s < .038, $\eta_{p}^{2}$_full_ = .006, $\eta_{p}^{2}$_reduced_ = .005. There were no significant differences on the *C* parameter, *F*s < 0.62, *p*s > .433, $\eta_{p}^{2}$_full_ = .001, $\eta_{p}^{2}$_reduced_ = .001, and *A* parameter, *F*s < 0.36, *p*s > .551, $\eta_{p}^{2}$_full_ < .001, $\eta_{p}^{2}$_reduced_ < .001.

# References

Cohen, J. (1988). *Statistical power analysis for the behavioral sciences* (2nd ed). Lawrence Erlbaum Associates, Publishers. https://doi.org/10.4324/9780203771587

Gawronski, B., Conway, P., Hütter, M., Luke, D. M., Armstrong, J., & Friesdorf, R. (2020). On the validity of the CNI model of moral decision-making: Reply to Baron and Goodwin (2020). *Judgment and Decision Making*, *15*(6), 1054–1072.

Liu, C., & Liao, J. (2021). CAN algorithm: An individual level approach to identify consequence and norm sensitivities and overall action/inaction preferences in moral decision-making. *Frontiers in Psychology*, *11*, 547916. https://doi.org/10.3389/fpsyg.2020.547916

# Table S1

Means and 95% confidence intervals of estimated CNI model parameters after excluding five potentially problematic dilemmas, pooled data from Experiments 1 to 3.

|  | **No Reason** | |  | **Think about Reasons** | |
| --- | --- | --- | --- | --- | --- |
|  | ***M*** | **95% CI** |  | ***M*** | **95% CI** |
| **Full Sample (*N* = 917)** |  |  |  |  |  |
| *C* parameter | .25 | [.23, .26] |  | .25 | [.23, .27] |
| *N* parameter | .64 | [.63, .66] |  | .67 | [.65, .69] |
| *I* parameter | .59 | [.57, .61] |  | .60 | [.57, .63] |
| **Reduced Sample (*N* = 869)** |  |  |  |  |  |
| *C* parameter | .25 | [.24, .26] |  | .25 | [.24, .27] |
| *N* parameter | .64 | [.62, .66] |  | .67 | [.65, .69] |
| *I* parameter | .58 | [.56, .61] |  | .60 | [.57, .64] |

*Note.* Full sample refers to the pooled data before exclusion of response-time outliers; reduced sample refers to the pooled data after exclusion of response-time outliers. CNI model parameter scores can range from 0 to 1.

# Table S2

Means and 95% confidence intervals of NCI model parameters, pooled data from Experiments 1 to 3.

|  | **No Reasons** | |  | **Think about Reasons** | |
| --- | --- | --- | --- | --- | --- |
|  | ***M*** | **95% CI** |  | ***M*** | **95% CI** |
| **Full Sample (*N* = 917)** |  |  |  |  |  |
| *C* parameter | .45 | [.43, .47] |  | .48 | [.46, .50] |
| *N* parameter | .41 | [.40, .42] |  | .45 | [.44, .46] |
| *I* parameter | .54 | [.52, .55] |  | .54 | [.52, .56] |
| **Reduced Sample (*N* = 869)** |  |  |  |  |  |
| *C* parameter | .45 | [.43, .47] |  | .48 | [.46, .50] |
| *N* parameter | .41 | [.40, .42] |  | .45 | [.43, .46] |
| *I* parameter | .53 | [.52, .55] |  | .54 | [.52, .56] |

*Note.* Full sample refers to the pooled data before exclusion of response-time outliers; reduced sample refers to the pooled data after exclusion of response-time outliers. NCI model parameter scores can range from 0 to 1.

# Table S3

Means and 95% confidence intervals of CAN algorithm parameters, pooled data from Experiments 1 to 3.

|  | **No Reasons** | |  |  | **Think about Reasons** | |
| --- | --- | --- | --- | --- | --- | --- |
|  | ***M*** | **95% CI** |  |  | ***M*** | **95% CI** |
| **Full Sample (*N* = 917)** |  |  |  |  |  |  |
| *C* parameter | .26 | [.25, .28] |  |  | .26 | [.24, .28] |
| *N* parameter | .41 | [.39, .43] |  |  | .45 | [.42, .48] |
| *A* parameter | .49 | [.48, .49] |  |  | .49 | [.48, .50] |
| **Reduced Sample (*N* = 869)** |  |  |  |  |  |  |
| *C* parameter | .26 | [.25, .28] |  |  | .26 | [.25, .28] |
| *N* parameter | .41 | [.39, .44] |  |  | .45 | [.42, .47] |
| *A* parameter | .49 | [.48, .49] |  |  | .49 | [.48, .50] |

*Note.* Full sample refers to the pooled data before exclusion of response-time outliers; reduced sample refers to the pooled data after exclusion of response-time outliers.

# Table S4

Results of the analyses of covariance conducted with the CAN algorithm parameters, pooled data from Experiments 1 to 3.

|  | **Full Sample (*N* = 917)** | | |  | **Reduced Sample (*N* = 869)** | | |
| --- | --- | --- | --- | --- | --- | --- | --- |
| **Model** | ***F*** | ***p*** | $\boldsymbol{\eta}_{\boldsymbol{p}}^{\boldsymbol{2}}$ |  | ***F*** | ***p*** | $\boldsymbol{\eta}_{\boldsymbol{p}}^{\boldsymbol{2}}$ |
| **Predicting *C* parameter** |  |  |  |  |  |  |  |
| *N* parameter | 145.50 | .000 | .137 |  | 134.73 | .000 | .135 |
| *A* parameter | 49.94 | .000 | .052 |  | 47.24 | .000 | .052 |
| Condition | 0.59 | .444 | .001 |  | 0.62 | .433 | .001 |
|  |  |  |  |  |  |  |  |
| **Predicting *N* parameter** |  |  |  |  |  |  |  |
| *C* parameter | 145.50 | .000 | .137 |  | 134.73 | .000 | .135 |
| *A* parameter | 48.51 | .000 | .050 |  | 49.37 | .000 | .054 |
| Condition | 5.79 | .016 | .006 |  | 4.31 | .038 | .005 |
|  |  |  |  |  |  |  |  |
| **Predicting *A* parameter** |  |  |  |  |  |  |  |
| *C* parameter | 49.94 | .000 | .052 |  | 47.24 | .000 | .052 |
| *N* parameter | 48.51 | .000 | .050 |  | 49.37 | .000 | .054 |
| Condition | 0.36 | .551 | .000 |  | 0.10 | .754 | .000 |

*Note.* Full sample refers to the pooled data before exclusion of response-time outliers; reduced sample refers to the pooled data after exclusion of response-time outliers.
